# Targeted exome sequencing reveals distinct pathogenic variants in Iranians with colorectal cancer

**DOI:** 10.18632/oncotarget.13977

**Published:** 2016-12-16

**Authors:** Hassan Ashktorab, Pooneh Mokarram, Hamed Azimi, Hasti Olumi, Sudhir Varma, Michael L. Nickerson, Hassan Brim

**Affiliations:** ^1^ Department of Medicine and Cancer Center, Howard University College of Medicine, Washington, DC, USA; ^2^ Department of Pathology, Howard University College of Medicine, Washington, DC, USA; ^3^ Hithru LLC, Silver Spring, MD, USA; ^4^ Laboratory of Translational Genomics, National Cancer Institute, Bethesda, MD, USA; ^5^ Current address: Department of Biochemistry, Shiraz University of Medical Sciences, Shiraz, Iran

**Keywords:** targeted exome sequencing, colon, Iranian, Shirazi, Caucasian

## Abstract

**PURPOSE:**

Next Generation Sequencing (NGS) is currently used to establish mutational profiles in many multigene diseases such as colorectal cancer (CRC), which is on the rise in many parts of the developing World including, Iran. Little is known about its genetic hallmarks in these populations.

**AIM:**

To identify variants in 15 CRC-associated genes in patients of Iranian descent.

**RESULTS:**

There were 51 validated variants distributed on 12 genes: 22% *MSH3* (*n* = 11/51), 10% *MSH6* (*n* = 5/51), 8% *AMER1* (*n* = 4/51), 20% *APC* (*n* = 10/51), 2% *BRAF* (*n* = 1/51), 2% *KRAS* (*n* = 1/51), 12% *PIK3CA* (*n* = 6/51), 8% *TGFβR2A* (*n* = 4/51), 2% *SMAD4* (*n* = 1/51), 4% *SOX9* (*n* = 2/51), 6% *TCF7L2* (*n* = 3/51), and 6% *TP53* (*n* = 3/51). Most known and distinct variants were in mismatch repair genes (*MMR*, 32%) and *APC* (20%). Among oncogenes, *PIK3CA* was the top target (12%).

**MATERIALS AND METHODS:**

CRC specimens from 63 Shirazi patients were used to establish the variant' profile on an Ion Torrent platform by targeted exome sequencing. To rule-out technical artifacts, the variants were validated in 13 of these samples using an Illumina NGS platform. Validated variants were annotated and compared to variants from publically available databases. An *in-silico* functional analysis was performed. MSI status of the analyzed samples was established.

**CONCLUSION:**

These results illustrate for the first time CRC mutational profile in Iranian patients. *MSH3*, *MSH6*, *APC* and *PIK3CA* genes seem to play a bigger role in the path to cancer in this population. These findings will potentially lead to informed genetic diagnosis protocol and targeted therapeutic strategies.

## INTRODUCTION

Colorectal cancer (CRC) is the third most common malignancy in the World and a significant contributor to cancer mortality and morbidity, including in Iran [1, 2]. Despite advances in early detection and therapies, it still has a lethal outcome in nearly 40% of all diagnosed cases [1, 3, 4]. CRC has multiple underlying genetic variants that associate with different clinical and pathological features [5–9].

Driver and recurrent mutational targets in CRC have been identified [10–12]. Driver mutations are generally responsible for triggering and promoting cancer development [11, 13]. Genetically, CRCs are either within the microsatellite instability category (MSI) (~15%) that are generally proximal and frequently associate with the CpG island methylator phenotype (CIMP) and hyper-mutation, or within the microsatellite stable (MSS) but chromosomally instable (CIN) prevalent category (~85%) [9, 14–17].

DNA mismatch repair (MMR) system consists of 6 proteins (MLH1, MSH2, MSH3, MSH6, PMS2 and PMS6) whose function is to repair DNA mismatches generated during replication. The Cancer Genome Atlas (TCGA) project reported *MSH3* variants in 40% of hypermutated tumors of which 3/4 were MSI-H [18–24]. The *MSH3* gene, located on chromosome 5q11–q12 [24, 25], encodes the MSH3 protein that has a partially redundant function with *MSH6* [25, 26]. Loss of *MSH3* has been reported in tumors with the Elevated Microsatellite instability At Selected Tetranucleotides repeats (EMAST) phenotype with instability at tetranucleotide repeats and poor prognosis [27].

We and others have previously reported the primary involvement of *MLH1* and *MSH2* alterations in MSI-H phenotype occurrence [28, 29]. However, loss of *MSH3* and *MSH*6 function was also cited with tumors arising in the right-colon that are poorly differentiated, mucine producing and generally with poor prognosis [30, 31].

*APC* is one of the key tumor suppressor genes (TSG) in the initiation of polyp formation [32] in both FAP and FAP-like sporadic CRCs [33]. *APC*'s role for downstream signaling with B-catenin, GSK and AXIN has been well documented [34].

Several studies have suggested that chromosome 18q loss is a critical event during CRC progression and that *SMAD4* gene is the primary target for inactivation [35]. Clinical experiments have shown that patients retaining heterozygosity at the 18q locus benefit significantly from treatment with 5-fluorouracil than patients with loss of heterozygosity at this site [36]. Also, most MSI CRCs display mutations at a microsatellite sequence in the transforming growth factor β receptor II gene (*TGFβR2A*) [37] which is involved in cell adhesion, cell migration and cell to cell communication. *PIK3CA*(phosphatidylinositol-3,4-bisphosphonate 3-kinase, catalytic subunit alpha) encodes the catalytic p110-alpha subunit of Phosphatidylinositol 3-Kinase (PI3K) alpha, which coordinates cell responses including cell proliferation, survival, proliferation, migration and morphology [38]. Activating *PIK3CA* variants are observed in various malignancies including CRC. *PIK3CA* variants are present in 10 to 15% of CRCs [38] and contribute to significant decrease of survival in patients with wild-type *BRAF* tumors [39].

*BRAF* oncogene is the key step in a malignant transformation within the methylation pathway to CRC [40]*. BRAF* gene is mutated in 4 to 12% of CRCs, more so in MSI tumors and in premalignant lesions, such as serrated adenomas and hyperplastic polyps [28, 41, 42]. *BRAF* mutations in CRC are associated with distinct clinical characteristics and worse prognosis [40].

In this study, we determined the frequency of variants present in CRC tissues of Iranian Caucasians. We report known and distinct validated variants in 12 genes using targeted exome sequencing. An *in-silico* functional analysis of these variants was performed.

## RESULTS

### Distinct pathogenic variants in CRC Shirazi specimens

#### MMR genes' variants

##### MSH3

**Figure 1:**
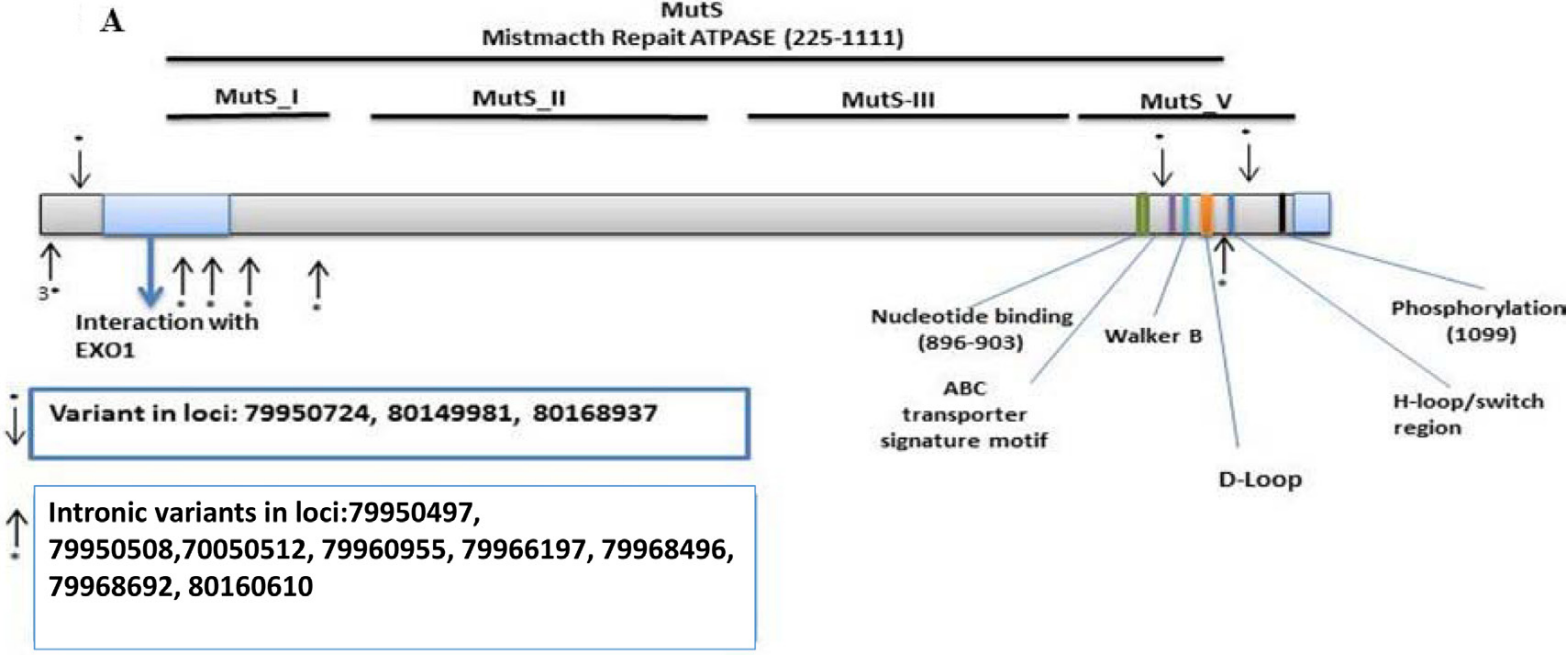

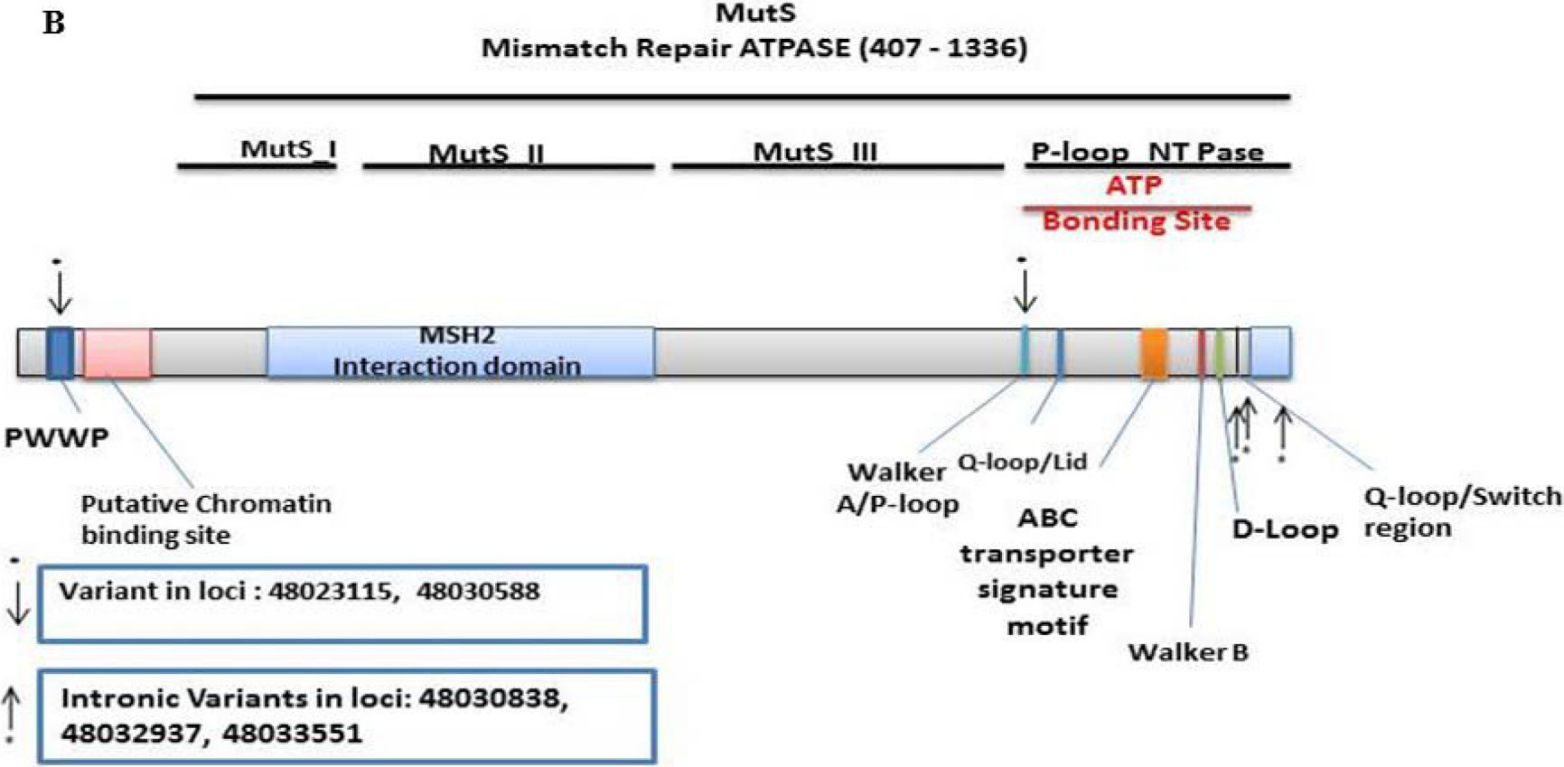
Distribution of validated variants per targeted genes (**A**) *MSH3* (**B**) *MSH6.*

We found 193 variants in the discovery set, of which 165 were distinct/novel. Of these novel variants, 66 were non-synonymous, 10 were stopgain, 23 were synonymous, and 94 were flanking intronic. The Illumina platform sequencing led to the validation of 11 known variants (Supplementary Table S1). From these, 3 were non-synonymous, variant at loci 79950724 with a G to C change in the MutS_I domain with a frequency of 0.02 (1/63, heterozygous), variant at loci 80149981 with an A to G change in the MutS_V domain with a frequency of 0.60 (38/63, 27 homozygous and 11 heterozygous), and variant at loci 80168937 with a G to A change in the MutS_V domain with a frequency of 0.51 (32/63, 20 homozygous and 12 heterozygous). The variants were mapped to the MSH3-MSH2-MSH6 region with 4 prior to EXO1, 2 in EXO1, 2 in MutS_I, and 3 in MutS_V (Figure 1A).

##### MSH6

There were 161 *MSH6* detected variants of which 139 were distinct. Of these, 82 were non-synonymous, 11 were stopgain, 22 were synonymous, and 24 were flanking intronic. The Illumina sequencing led to the validation of 5 known variants (Supplementary Table S2). From these, 1 was synonymous at loci 48023115 with a T to C change at MutS_II with a frequency of 0.14 (9/63, 3 homozygous and 6 heterozygous), and 1 stopgain at loci 48030588 with a C to T change at MutS_II with a frequency of 0.06 (4/63, all heterozygous). The other 3 were intronic. One variant was mapped in PWWP, and 4 in the P-loop N_TPase region (Figure 1B).

### CRC-associated tumor suppressor genes' variants

#### AMER1

**Figure 2:**
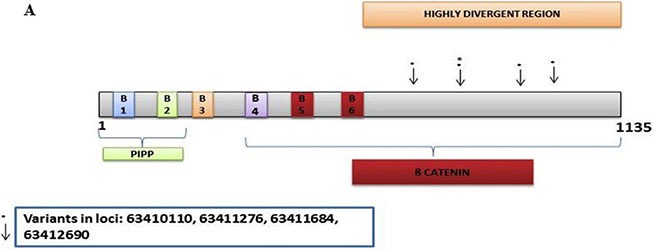

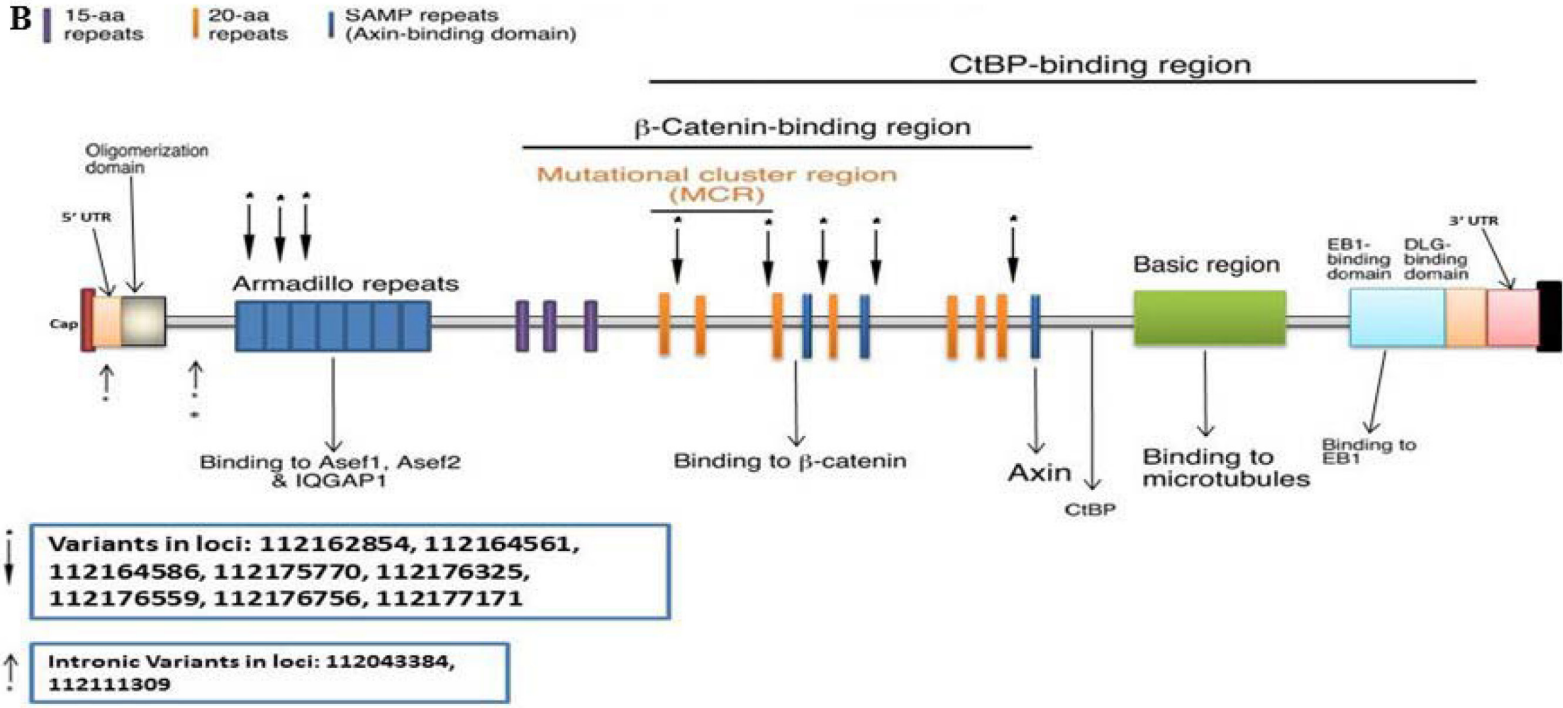

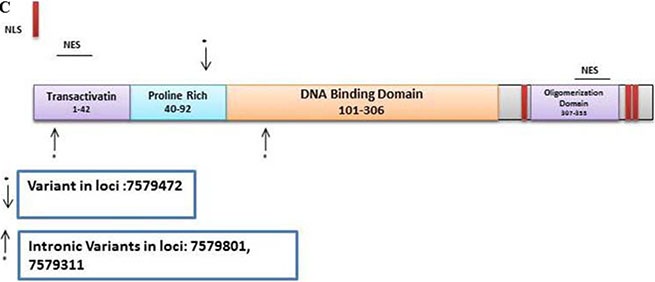

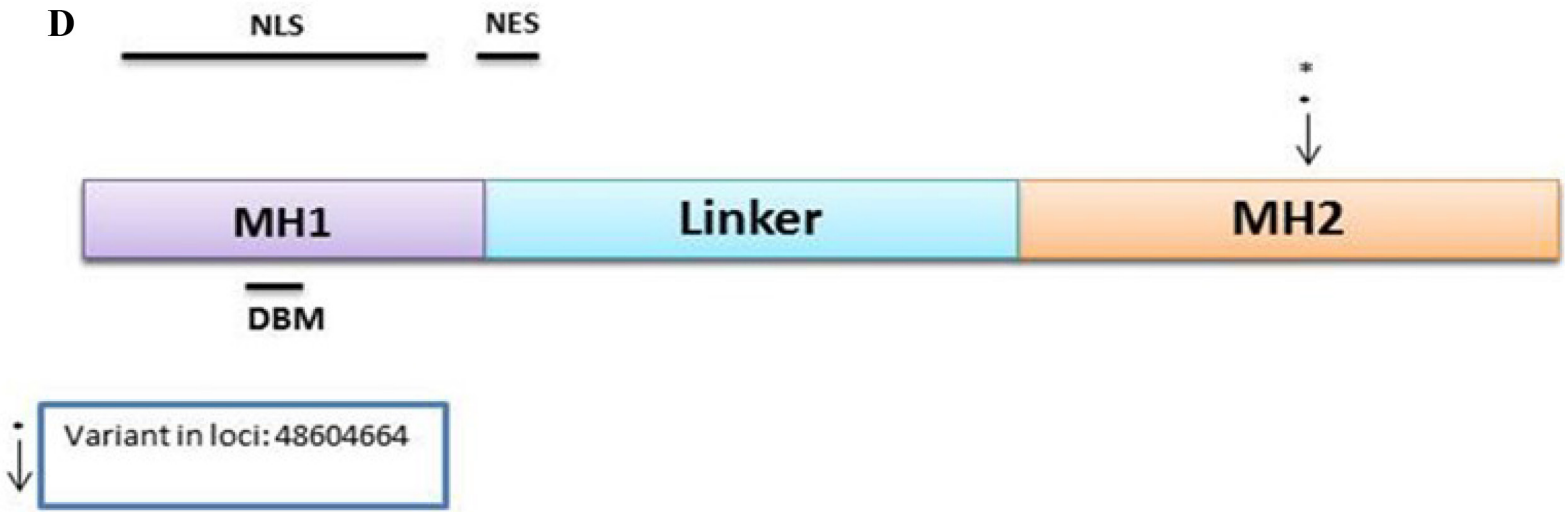

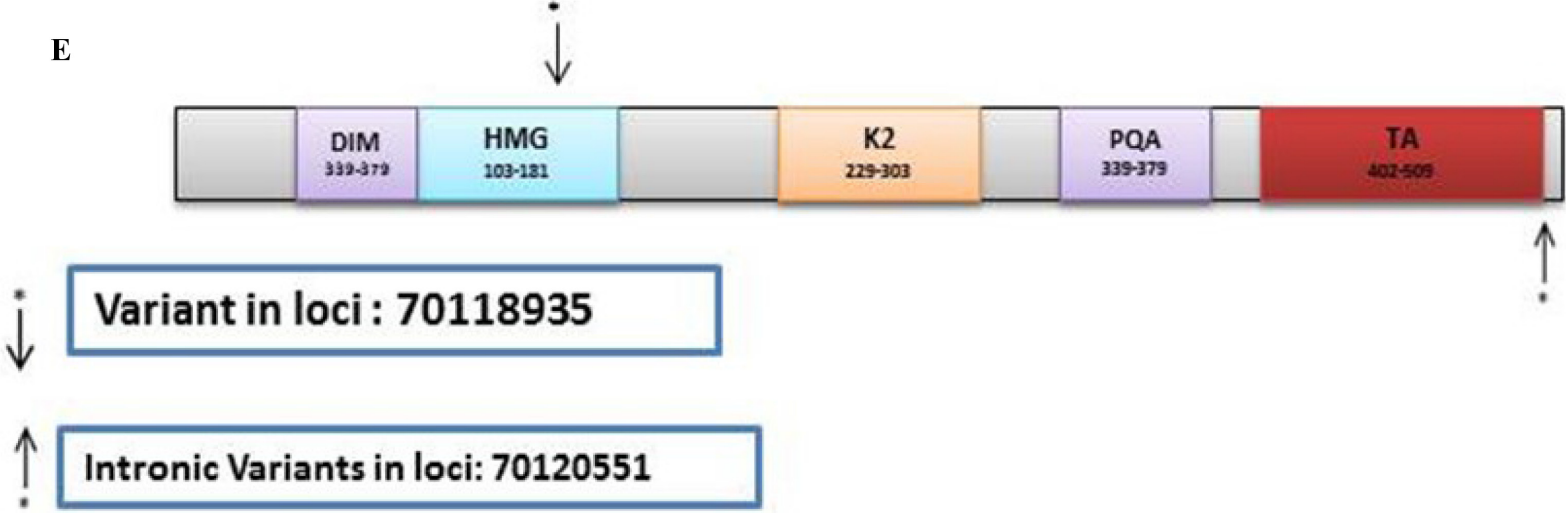

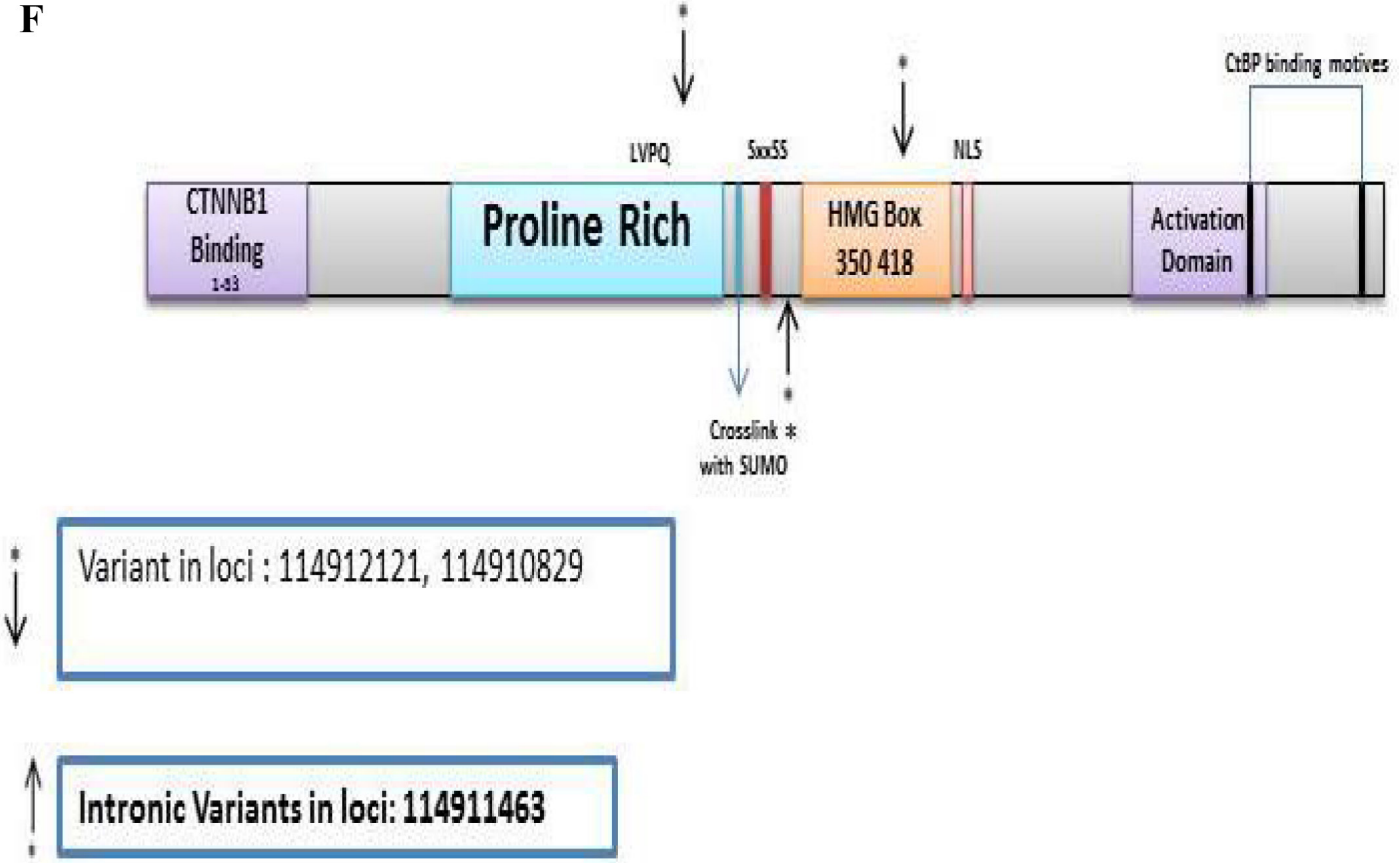

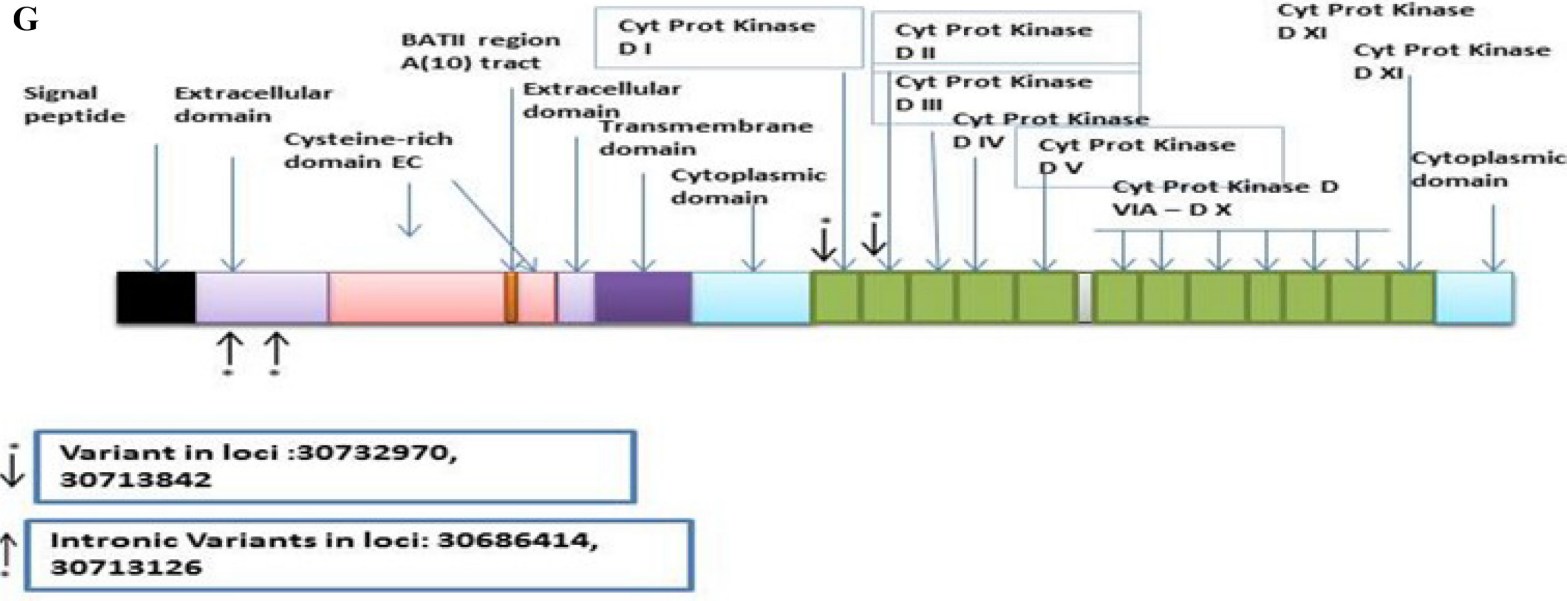

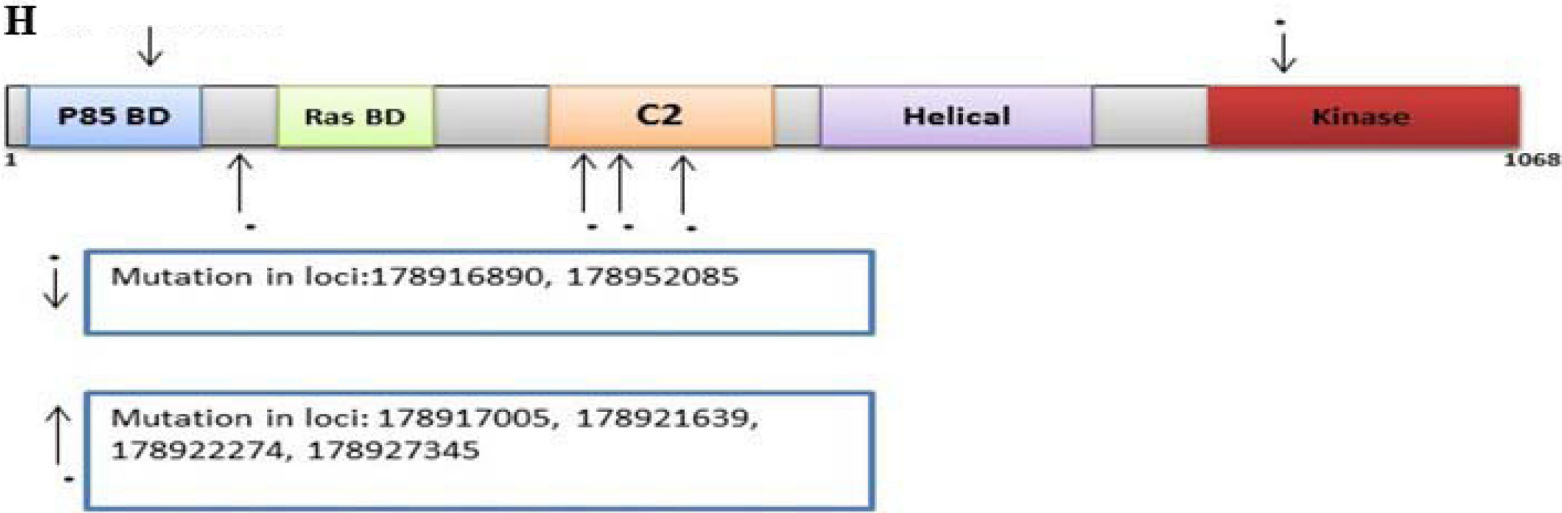

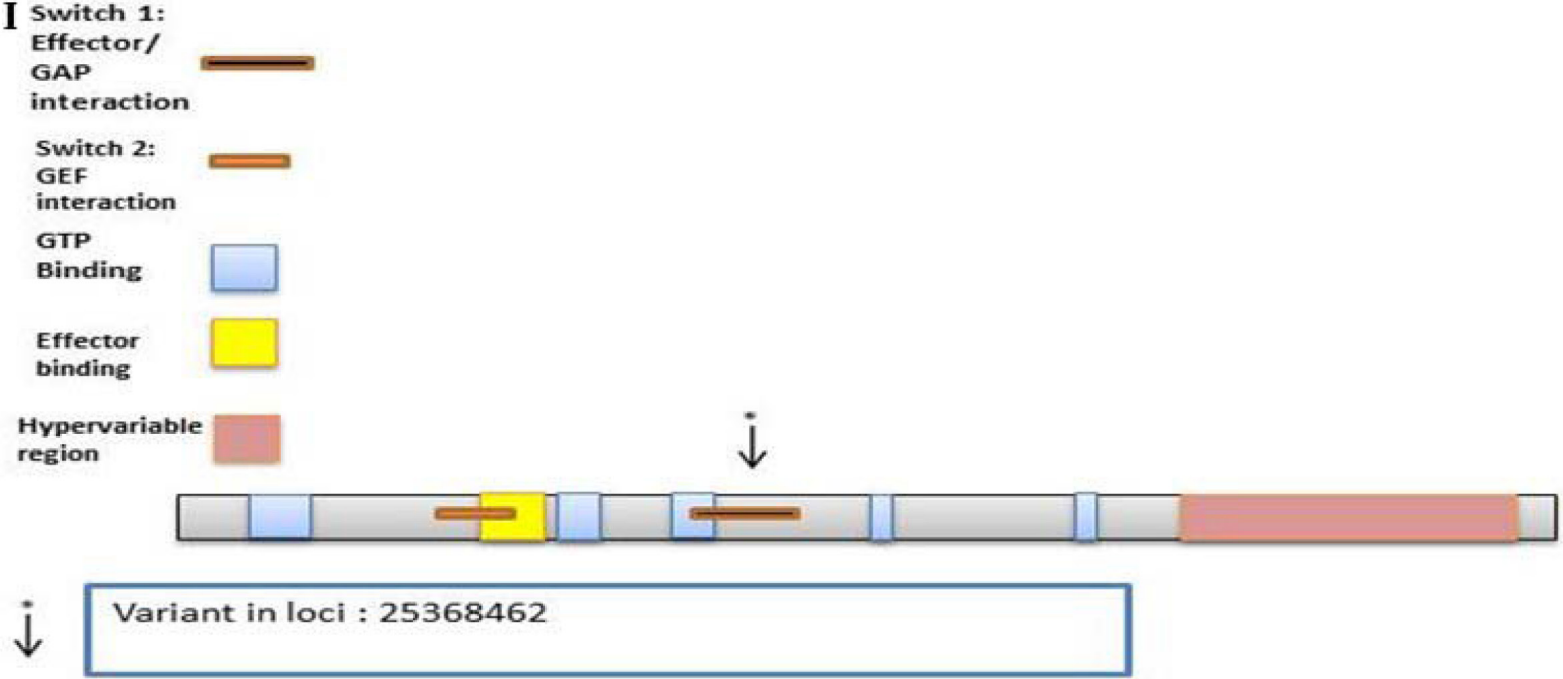

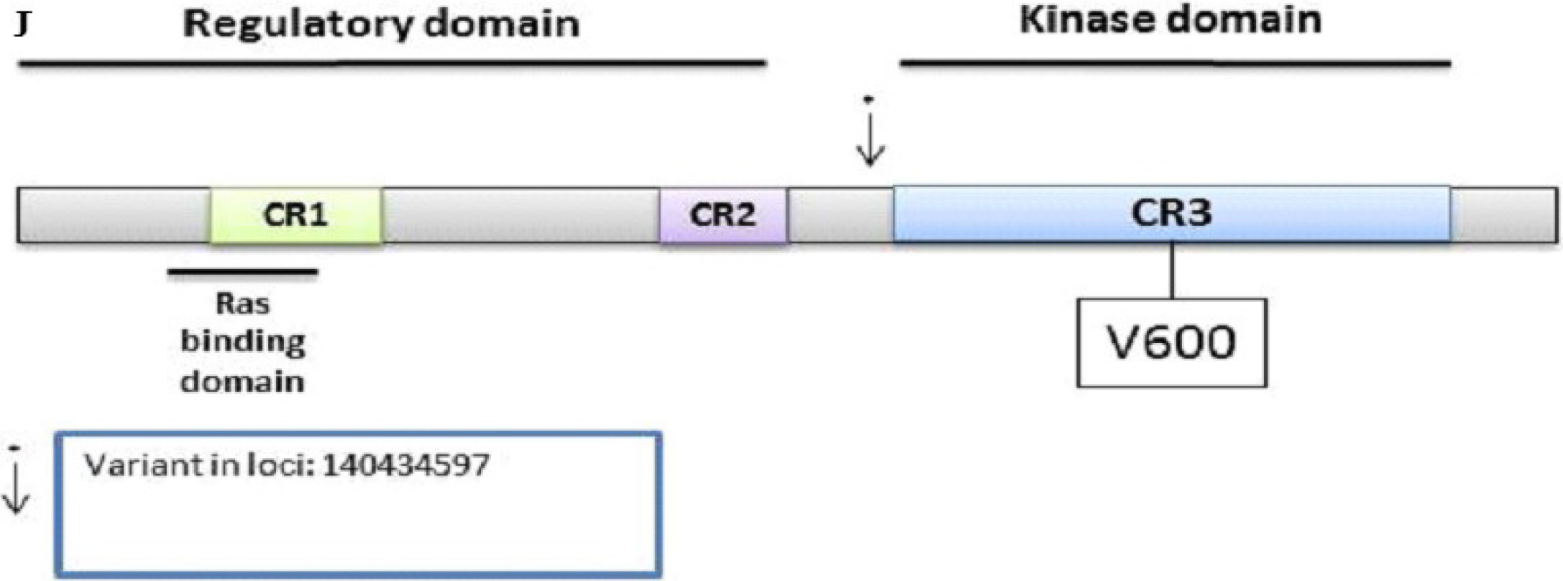
CRC Associated genes (**A**) *AMER1,* **(B)** *APC,* **(C)** *TP53,* **(D)** *SMAD4,* **(E)** *SOX9,* **(F)** *TCF7L2,* **(G)** *TGFβR2A,* **(H)** *PIK3CA,* **(I)** *KRAS,* **(J)** *BRAF*.

In the discovery set there were 167 variants detected, with 155 novel. Of these, 103 were non-synonymous, 12 were stopgain, 32 were synonymous, and 8 were intronic. The Illumina sequencing led to the validation of 4 variants of which 1 was novel/distinct (Supplementary Table S3). The novel variant at loci 63411684 exon 2 was non-synonymous with a G to T change with a frequency of 0.06 (4/63, all heterozygous). The other non-synonymous variant at loci 63412690 had an A to C change with a frequency of 0.06 (4/63, 2 homozygous and 2 heterozygous). The stopgain variant at loci 63411276 had a G to A change with a frequency of 0.06 (4/63, all heterozygous). The synonymous variant at loci 63410110 had a T to C change with a frequency of 0.08 (5/63, 1 homozygous and 4 heterozygous). All 4 variants were mapped in the highly divergent region (Figure 2A).

#### APC

There were 352 variants in the discovery set with 305 distinct. From these, 169 were non-synonymous, 30 were stopgain, 65 were synonymous, and 41 were intronic. The Illumina sequencing led to the validation of 10 variants of which 1 was novel/distinct (Supplementary Table S4). From these 10 variants, 1 was stopgain, 1 non-synonymous, 6 were synonymous, and 2 were intronic. The stopgain variant at loci 112164586 had a C to T change with a frequency of 0.06 (4/63, all heterozygous). The non-synonymous variant at loci 112176756 had a T to A change with a frequency of 0.57 (36/63, 27 homozygous and 9 heterozygous). One variant was mapped in the 5' UTR, 1 prior to the ARM, 3 in the ARM, and 5 in the β-Catenin-binding region (Figure 2B).

#### TP53

There were 106 variants in the discovery set with 61 novel. From these, there were 13 non-synonymous, 1 was stopgain, 5 were synonymous and 42 were intronic variants. The Illumina platform sequencing led to the validation of 3 known variants (Supplementary Table S5). One was non-synonymous at loci 7579472 with a G to C change with a frequency of 0.40 (25/63, all heterozygous) and mapped to the Proline rich region. The 2 intronic variants at loci 7579801 (exonic in 3 TP53 transcripts) with a frequency of 0.37 (23/63, all heterozygous) and loci 7579311 (intronic for all TP53 transcripts) with a frequency of 0.05 (3/63, all heterozygous) were mapped in the transactivation and DNA Binding Domain, respectively (Figure 2C).

#### SMAD4

The discovery set led to 129 variants, of which 113 were distinct. From these, 56 were non-synonymous, 7 were stopgain, 11 were synonymous, and 39 were intronic. The Illumina platform sequencing led to the validation of 1 novel variant (Supplementary Table S6). This non-synonymous variant at loci 48604664 exon 12 with a C to T change with a frequency of 0.08 (5/63, all heterozygous) was mapped in the MH2 domain (Figure 2D).

### CRC-associated Oncogenes' variants

#### SOX9

In the discovery set, there were 85 variants of which 77 were novel. Of these, 34 were non-synonymous, 5 were stopgain, 26 were synonymous, and 12 were intronic. The Illumina sequencing led to the validation of 2 known variants (Supplementary Table S7). From these, one was synonymous at loci 70118935 with a C to T change with a frequency of 0.13 (8/63, all heterozygous) mapped in the HMG region, and one was intronic at loci 70120551 with A to C change with a frequency of 0.35 (22/63, 5 homozygous and 17 heterozygous) mapped after the TA region (Figure 2E).

#### TCF7L2

There were 153 variants of which 141 were novel in the discovery set. From these, 46 were non-synonymous, 4 were stopgain, 15 were synonymous, and 76 were intronic. The Illumina sequencing led to the validation of 3 variants of which 1 was novel/distinct (Supplementary Table S8). Two synonymous variants, at loci 114912121 with a G to A change with a frequency of 0.08 (5/63, all heterozygous), and loci 114910829 with an A to G change with a frequency of 0.05 (3/63, all heterozygous) and were mapped to the proline rich region and HMG Box, respectively. The 1 intronic variant was novel at loci 114911463 with a G to A change with a frequency of 0.06 (4/63, all heterozygous) and mapped prior to the HMG box (Figure 2F).

#### TGFβR2A

The discovery set led to 118 variants of which 107 were distinct. Of these, 48 were non-synonymous, 3 were stopgain, 21 were synonymous, and 35 were intronic (Supplementary Table S10). The Illumina platform sequencing led to the validation of 4 known variants. From these, 1 was non-synonymous, 1 synonymous, and 2 intronic. The non-synonymous variant at loci 30732970 with a G to A change with a frequency of 0.06 (4/63, all heterozygous) was mapped to the PKinase domain region. The synonymous variant at loci 30713842 with a C to T change with a frequency of 0.02 (1/63, heterozygous) was also mapped to the PKinase domain region. The 2 intronic variants were mapped in the extracellular domain (Figure 2G).

#### PIK3CA

There were 157 variants in the discovery set, with 128 distinct. Of these, 57 were non-synonymous, 8 were stopgain, 14 were synonymous, and 49 were intronic. The Illumina platform sequencing led to the validation of 6 known variants of which 2 were non-synonymous and 4 intronic (Supplementary Table S9). One non-synonymous variant at loci 178916890 had a C to T change with a frequency of 0.06 (4/63, all heterozygous). The other non-synonymous variant at loci 178952085 had an A to G change with a frequency of 0.06 (4/63, all heterozygous). One variant was mapped in the P85 BD region, 1 prior to Ras BD, 4 within the C2 region, and 1 in the Kinase region (Figure 2H).

#### KRAS

There were 33 variants on the discovery set of which 23 were novel. From these, 9 were nonsynonymous, 2 were synonymous, and 12 were intronic. The Illumina sequencing led to the validation of 1 known variant (Supplementary Table S11). It was synonymous at loci 25368462 with C to T substitution with a frequency of 0.67 (42/63, 40 homozygous and 2 heterozygous) in the RAS domain region (Figure 2I).

#### BRAF

We detected 139 variants on the Ion Torrent platform, of which 120 were distinct. In this set, 44 were non-synonymous, 4 were stopgain, 19 were synonymous, and 53 were intronic (Supplementary Table S12). The Illumina platform sequencing led to the validation of 1 known variant. This variant was intronic at loci 140434597 with a G to A change with a frequency of 0.16 (10/63, all heterozygous) and mapped before the Kinase domain (Figure 2F).

#### MSI status

In the discovery set, 25% (*n* = 16/63) of tumors were MSI-H (2 on the left side and 14 on the right side), (Table 1). The two MSI-H tumors on the left side were in stage I male patients. For the MSI-H tumors on the right side, there were 6 females (stage II) and 8 males (2 stage I, 4 stage II and 2 stage III). Also 10% (6/63) of tumors were MSI-L while the remaining 65% (41/63) were MSS. In the validated set, 38% (*n* = 5/13) were MSI-H (all on the right side; Table 1). Three tumors were on the left side, all 3 were stage I. There were 10 tumors on the right side, of which 4 were stage I, 5 were stage II, and 1 was stage III. The MSI-H tumors on the right side were classified as 1 stage I, 3 stage II, and 1 stage III.

**Table 1:** Clinico-pathological characteristics and variants' (novel and known) distribution in the validation set

Sample ID	Sex	Age	Stage	MSI	Location	MSH3	MSH6	APC	PIK3CA	TGF0R2A	SMAD4	AMER1	KRAS	TCF7L2	SOX9	p53
CC1759	F	55	II	MSI-H	R	+	−	−	−	+	−	+	−	−	−	−
CC1760	M	50	I	MSS	L	+	−	−	−	+	−	−	−	−	−	−
CC1762	F	55	I	MSS	R	+	−	−	−	+	−	−	−	−	−	−
CC1764	M	63	I	MSS	L	+	−	+	+	+	−	+	−	+	−	−
CC1765	M	43	I	MSI-H	R	+	+	+	+	+	+	+	+	+	+	+
CC1817	M	77	III	MSI-H	R	+	+	+	+	+	+	+	+	+	+	+
CC1818	M	50	II	MSI-H	R	+	+	+	+	+	+	+	+	+	+	+
CC1819	M	70	II	MSI-H	R	+	+	+	+	+	+	+	+	+	+	+
CC1820	M	52	II	MSS	R	+	+	+	+	+	−	−	+	−	+	+
CC1821	M	62	II	MSS	R	+	+	+	+	+	+	−	+	+	−	−
CC1822	F	55	I	MSS	L	+	+	+	+	+	−	+	+	+	+	+
CC1823	F	60	I	MSS	R	+	+	+	+	+	−	+	+	+	+	+
CC1824	F	58	I	MSS	R	+	+	+	+	+	−	+	+	+	+	+

+ = Presence of variant includes distinct and known listed genes, − =Absence of variant.

## DISCUSSION

Targeted exome sequencing led to variants' profile of driver genes in Iranian Caucasians with sporadic CRC that resulted in the validation of 51 known and distinct variants. While we reported hundreds of variants in the results section, we will only discuss the 51 validated ones.

At first glance, the validated variants seem to be more in MMR and TSGs combined than in oncogenes. Indeed, 32% were in MMR genes [22% *MSH3 (n = 11/51)*, 10% *MSH6 (n = 5/51)*], 36% were in TSGs [8% *AMER1 (n = 4/51),* 20% *APC (n = 10/51)*, 6% *TP53 (n = 3/51)*, and 2% *SMAD4 (n = 1/51),* while 34% were in oncogenes [*4*% *SOX9 (n = 2/51),* 6% *TCF7L2 (n = 3/51),* 8% *TGFβR2A (n = 4/51), 12% PIK3CA (n = 6/51),* 2% *KRAS (n = 1/51)*, and 2% *BRAF (n = 1/51*]. The most frequent known and distinct variants were in mismatch repair genes (MMR, 32%) and *APC* (20%). Among oncogenes, *PIK3CA* was the top target with 12% of validated variants. While this description already provides the big picture of the important relevance of MMR and APC in CRC pathogenesis in the analyzed population, specific description of the detected variants as far as the nature of variant, homozygous vs. heterozygous status and frequency within the targeted population is warranted for a more precise assessment of the variants and genes' weight in Iranian CRC patients.

DNA MMR protein heterodimers interact in a series of steps that include the association of MSH2 with either MSH3 or MSH6 to form MutSβ or MutSα complexes, respectively [19, 30, 43, 44]. Our distinct validated variants in *MSH3* and *MSH6* result in a defective DNA MMR based on *in-silico* functional analysis. It appears to suggest altered static interactions within the MSH2-MSH3 and the MSH2-MSH6 heterodimers [29, 45]. Our distinct variants for the MSH3 and MSH6 are at the sites of MSH2 binding, and these variants are likely to disturb the complexes formed by MSH3 and MSH6 with MSH2. The Cancer Genome Atlas (TCGA) project reported that *MSH3* variants were spotted in 40% of hypermutated CRC tumors of which 3/4 exhibited MSI-H [24]. This is consistent with our cohort's pathological features since MSI-H rate was high as well (25%).

There were 3 non-synonymous variants for *MSH3,* 1 in the MutS_I domain and 2 in the MutS_V region (Figure 1A) which are on the binding site for the MSH3-MSH2 which likely alters the mismatch repair system and affect the MutSβ complex [46]. We also found 8 variants in splice sites in *MSH3*, many of which may lead to loss of protein function through abnormal splicing.

MSH6 has a domain with intrinsic ATPase activity [30]. Variants within this region have previously been shown to affect the mismatch repair system in the progression to CRC [30]. Variants at loci 48023115 at Mut_II and at loci 48030588 at MutS_II with a frequency of 0.06 mapped to MSH2 interaction domain of *MSH6* (Figure 1B). These variants likely lead to loss of function of the MutS complex [47]. Most of the samples with both *MSH6* and *MSH3* variants are at advanced stage and were proximal. This is consistent with reports of *MSH3* defects' association with EMAST phenotype and poor prognosis [27, 46]. In addition, MSH3 function in double strand break repair and homologous recombination is partially responsible for sensibility to drugs such as 5FU and Oxaliplatin. Therefore, the validated variants may result in different response to therapy [48]. We also validated 3 variants at the *MSH3/DHFR* promotor region 43bp, 59bp and 104bp away from *MSH3* tsp/exon 1. It has been reported that variants in the *MSH3/DHFR* that associate with an amplification and overexpression of MSH3 lead to an imbalance of MutS-Alpha/MutS_Beta ratio that associates with reduced DNA repair activity [49]. The specific effects of the validated *MSH3/DHFR* variants in the path to CRC needs further investigation and analysis.

It is also worth noting that among the 3 *MSH3* validated non-synonymous variants [variant at loci 79950724/MutS_I domain, frequency of 0.02 (1/63, heterozygous), variant at loci 80149981/MutS_V domain, frequency of 0.60 (38/63, 27 homozygous and 11 heterozygous), and variant at loci 80168937/MutS_V domain, frequency of 0.51 (32/63, 20 homozygous and 12 heterozygous)], the MutS_V variants], two were frequent and highly homozygous in this population. This finding gives a special weight to these two variants as they are likely to increase the rate of the EMAST phenotype within the analyzed population. Such is not the case for *MSH6* variants that displayed lower frequencies and homozygosity [synonymous variant at loci 48023115/MutS_II, frequency of 0.14 (9/63, 3 homozygous and 6 heterozygous), and 1 stopgain at loci 48030588/MutS_II, frequency of 0.06 (4/63, all heterozygous)].

The *AMER1* gene had 4 validated variants including a validated synonymous one, were mapped in the highly divergent region (Figure 3A). Other exome studies have reported that *AMER1* is indeed a frequent target of mutation in colorectal cancer [50, 51]. The non-synonymous and stopgain variants, although heterozygous, had a frequency of 6% that is still fairly high in this population pointing to the relevance of this gene in the path to cancer.

Ten *APC* variants were validated of which 1 was stopgain, 1 non-synonymous, 6 were synonymous, and 2 were intronic. (Figure 2B). Of the validated variants, the non-synonymous one seems to have a special weight in Iranian CRC as it occurs in 57% of the analyzed patients and in 43% (27 out of 63) it is homozygous. This finding further strengthen the importance of this gene and the Wnt pathway in colon carcinogenic transformation in this group of patients.

We validated 3 known *TP53* variants. One was non-synonymous mapped to the Proline rich region. While this frequent variant (40%) did not exist in a homozygous state in any of the analyzed patients, the second allele might also be targeted by a different variant that will lead to a complete inactivation of TP53. Indeed, other validated variants [2 intronic variants at loci 7579801, frequency of 0.37 (23/63, all heterozygous) and loci 7579311, frequency of 0.05 (3/63, all heterozygous)] were also reported in this population (Figure 3E). While intronic variants are very unlikely to show up in the mature protein (except in cases of alternative splicing), such variants might affect the reading of the mRNA and generate abnormal proteins through the integration or deletion of genetic information from the mature protein [52–54].

As for *SMAD4* gene, one novel non-synonymous variant was mapped in the MH2 domain (Figure 2D). Several studies have reported mutations and variants within the mutational hotspot region MH2 region, with up to 80% of mutations in this gene within this region [35]. The heterozygous nature of the validated variant in our study, however, lowers its potential impact on the oncogenic transformation.

The oncogenes in this study displayed less variants when compared with tumor suppressor genes and DNA MMR genes combined. For *SOX*9, 2 known variants were validated: a synonymous at loci and an intronic at loci mapped after the TA region (Figure 2E). SOX9 was already reported to act on B-Catenin and PPARRgamma activation in colorectal cancer [55]. However, how the validated intronic variant might be involved in such a process remains to be elucidated.

For *TCF7L2*, there were 3 validated variants of which 1 was novel. (2 synonymous and 1 intronic variant). The intronic variant was mapped prior to the HMG box (Figure 2F). It would be interesting to functionally assess the potential role of this intronic variant in the generation of splice variants. Indeed, Nome et al. have already reported a high frequency of fusion transcripts of *TCF7L2* in colorectal cancer patients as a result of splicing alterations [56].

For *TGFβR2A*, there were 4 validated known variants. One was non-synonymous, 1 synonymous, and 2 intronic. The non-synonymous variant was mapped to the PKinase domain region which may affect kinase activity. (Figure 2G). Intronic variants within *TGFβR2* gene have already been reported in oral carcinoma as a result of aberrant splicing [57]. Such might be the case for the validated intronic mutations in the present study.

Among these, *PIK3CA* was the top targeted gene with 6 validated known variants of which 2 were non-synonymous and 4 intronic. One variant was mapped in the P85 BD region, 1 prior to Ras BD, 4 within the C2 region, and 1 in the Kinase region (Figure 2H) which may be important for the kinase activity. A functional *in-vitro* analysis of these validated variants is necessary to determine their potential effect on the protein activity.

For *KRAS*, only 1 known synonymous variant in the RAS domain region was validated (Figure 2I). The high prevalence of this variant (67%) with most of it in homozygous state points to either a polymorphism that is specific to the analyzed population or to a high mutational spot. We think that this variant likely points to a local polymorphism within the *KRAS* that is independent of colorectal cancer transformation.

As for *BRAF*, the only validated variant was intronic (known) with a higher prevalence than *PIK3CA* variants (16% vs. 6%). This variant was mapped before the Kinase domain (Figure 2J).

The majority of the samples analyzed through the TCGA project, as well as those deposited in 1000Genomes/dbSNP/COSSMIC databases, are from Caucasians living in the West. This might be one of the reasons of the novelty of the variants we described here. As such, there is a need to add these new cancer genes' variants along with others to highlight similarities and specifics in the World's CRC population. We would like to state that the novel variants germline or somatic status cannot be ascertained in the present study -even though patients with family history (HNPCC or FAP) were excluded from this study- because of a lack of matched normal tissue. More to the point, these variants classified as new since most of the TSG and Oncogenes variants have been compared with data in those in 1000 Genomes, dbSNP, TCGA and COSMIC databases. The introduced novel variants for highly studied genes such as *APC, MMR, KRAS, BRAF, PIK3CA,* and *P53* are unlikely to be present in the matched normal.

In conclusion, our study shows, for the first time in Iranian CRC patients, the importance of distinct and known pathogenic variants in colorectal cancer genes through targeted exome sequencing. *MSH3, APC, BRAF* and *PIK3CA* were the primary targets with a higher prevalence in the analyzed cohort. This will potentially lead to informed genetic diagnosis protocol and tailored therapeutic strategies in this population.

## MATERIALS AND METHODS

### Discovery set

CRC specimens (Supplementary Table S1) were collected from 63 Iranian, Shirazi patients from Shiraz Medical Sciences University, Iran, and used to establish the variants' profile by targeted exome sequencing on an Ion Torrent platform. Subjects with familial adenomatous polyposis (FAP), hereditary nonpolyposis (HNPPC), or a family history of CRC were excluded. The study was approved by the Institutional Review Board of Shiraz Medial Sciences University, and written informed consent was obtained from all patients. The summary characteristics for all patients are listed in Table 2. There were 25 (40%) females and 38 (60%) males. The age range was 40 to 82 with a median age of 60 years. Fifty-four percent *(n* = 34) of the patients were stage I, 43% (*n* = 27) were stage II, and 3% (*n* = 2) were stage III. More than half (*n* = 33, 52%) of the tumors were left sided.

**Table 2:** Clinico-pathological characteristics of patients in the discovery set

Sex	*n* = 63 (%)	
	Male	38 (60)
	Female	25 (40)
**Location**		
	Right	30 (48)
	Left	33 (52)
**Stage**		
	I	34 (54)
	II	27 (43)
	III	2 (3)
**MSI**		
	MSI-L	6 (10)
	MSI-H	16 (25)
	MSS	41 (65)

### Validation set

A subset of 13 cases (Table 1) from the original 63 samples were used for validation by targeted exome sequencing on a HiSeq platform (Illumina, San Diego, CA). The validated variants listed in Table 3. Genomic DNA from each patient's tissue sample was fragmented and hybridized to commercially available capture arrays for enrichment according to our previous study [7]. The characteristics for all patients are listed in Table 2. The age range was from 43 to 77 with a median age of 55 years. 54% (*n* = 7) of the patients were stage I, 38% (*n* = 5) were stage II, and 8% (*n* = 1) were stage III. There were 5 (38%) females and 8 (62%) males. There were 10 (77%) right sided tumors.

**Table 3:** Validated of distinct and known variants in mismatch repair, tumor suppressors and oncogenes

	Loci	Ref	Var	Gene	Variant type	Novel	Frequency of Mutation
***Mismatch Repair***							
	*80149981*	*A*	*G*	*MSH3*	*nonsynonymous SNV*	*0*	*0.60*
	*80168937*	*G*	*A*	*MSH3*	*nonsynonymous SNV*	*0*	*0.51*
	*79950724*	*G*	*C*	*MSH3*	*nonsynonymous SNV*	*0*	*0.02*
	*48023115*	*T*	*C*	*MSH6*	*synonymous SNV*	*0*	*0.14*
	*48030588*	*C*	*T*	*MSH6*	*stopgain*	*0*	*0.06*
***Tumor Suppressor***							
	*63410110*	*T*	*C*	*AMER1*	*synonymous SNV*	*0*	*0.08*
	*63412690*	*A*	*C*	*AMER1*	*nonsynonymous SNV*	*0*	*0.06*
	*63411276*	*G*	*A*	*AMER1*	*stopgain*	*0*	*0.06*
	*63411684*	*G*	*T*	*AMER1*	*nonsynonymous SNV*	*1*	*0.06*
	*112162854*	*T*	*C*	*APC*	*synonymous SNV*	*0*	*0.52*
	*112164561*	*G*	*A*	*APC*	*synonymous SNV*	*0*	*0.49*
	*112176325*	*G*	*A*	*APC*	*synonymous SNV*	*0*	*0.49*
	*112176559*	*T*	*G*	*APC*	*synonymous SNV*	*0*	*0.54*
	*112177171*	*G*	*A*	*APC*	*synonymous SNV*	*0*	*0.59*
	*112176756*	*T*	*A*	*APC*	*nonsynonymous SNV*	*0*	*0.57*
	*112175770*	*G*	*A*	*APC*	*synonymous SNV*	*0*	*0.49*
	*112164586*	*C*	*T*	*APC*	*stopgain*	*0*	*0.06*
	*7579472*	*G*	*C*	*TP53*	*nonsynonymous SNV*	*0*	*0.40*
	*48604664*	*C*	*T*	*SMAD4*	*nonsynonymous SNV*	*1*	*0.08*
***Oncogenes***							
	*70118935*	*C*	*T*	*SOX9*	*synonymous SNV*	*0*	*0.13*
	*114912121*	*G*	*A*	*TCF7L2*	*synonymous SNV*	*0*	*0.08*
	*114910829*	*A*	*G*	*TCF7L2*	*synonymous SNV*	*0*	*0.05*
	*30732970*	*G*	*A*	*TGFBR2*	*nonsynonymous SNV*	*0*	*0.06*
	*30713842*	*C*	*T*	*TGFBR2*	*synonymous SNV*	*0*	*0.02*
	*178916890*	*C*	*T*	*PIK3CA*	*nonsynonymous SNV*	*0*	*0.06*
	*178952085*	*A*	*G*	*PIK3CA*	*nonsynonymous SNV*	*0*	*0.06*
	*25368462*	*C*	*T*	*KRAS*	*synonymous SNV*	*0*	*0.67*

### Targeted sequencing and analysis methods by ion torrent

A targeted, multiplex PCR primer panel was designed using the custom Ion Ampliseq Designer v1.2 (Thermo Fisher Scientific). The panel amplified 56.9 kb and included the coding regions of 20 genes, with an average coverage of 96.9% of the protein coding regions and splice junctions. In this study, we only report data from the 15 genes that are common between Illumina and Ion Torrent gene panels. The panel was designed to amplify PCR products appropriate for use with DNA from formalin-fixed paraffin embedded (FFPE) tissue with an average amplicon size of 150 base pairs (bp). Sample DNA (20 ng/primer pool) was amplified using the primer panel, and libraries were prepared using the Ion Ampliseq Library Preparation kit following the manufacturer's protocol (Thermo Fisher Scientific, Grand Island, NY). Individual samples were barcoded, pooled, and sequenced on an Ion Torrent Proton Sequencer using the Ion PI Template OT2 200v3 and Ion PI Sequencing 200v2 kits per manufacturer's instructions. Raw sequencing reads were filtered for high quality reads, and the adaptors were removed using the Ion Torrent Suite 4.0.4, then reads were aligned to the hg19 reference sequence by TMAP (https://github.com/iontorrent/TS/tree/master/Analysis/TMAP) using default parameters. Resulting BAM files were processed through an in-house quality control (QC) filter and coverage analysis pipeline. BAM files were aligned using GATK LeftAlignIndels module. Amplicon primers were trimmed from aligned reads by Torrent Suite. Variant calls were made by Torrent Variant Caller 4.0 (http://mendel.iontorrent.com/ion-docs/Torrent-Variant-Caller-Plugin.html) and listed as Supplementary Data (S1) were described previously [58].

### Targeted exome sequencing reads alignment using illumina

Details regarding DNA quantification and quality assessment, for the validation set (Illumina sequencing platform), SNV calling, public genome data comparison, sequencing validation, SNV description, variant frequencies, and copy number alterations were described previously [6, 7, 58].

### Bioinformatics

An average sequencing depth of >1000x was achieved and >98% of targeted bases (coding and within 5 bp of intron-exon boundaries) were examined by >10 reads required for variant identification. Variants were annotated using ANNOVAR [59] and filtered using the 1000 Genomes database, which represents a nominally noncancerous population, and dbSNP build 138. In addition, variants were filtered using the COSMIC databases. Variants present in any of those three datasets were marked as non-novel (known). All samples displayed more or less an equal number of SNVs in their tumors compared with their matched normal samples.

### *In silico* functional analysis

Polymorphism data for the genes were retrieved from the following databases: The UniProt database (http://www.uniprot.org) (UniProtKB ID Q8IYM9), the NCBI dbSNP database (https://www.ncbi.nlm.nih.gov/SNP/), Catalogue Of Somatic Mutations In Cancer (COSMIC http://cancer.sanger.ac.uk/cosmic) and 1000 Genomes (http://www.1000genomes.org/). Variants present in dbSNP, 1000 Genomes or COSMIC were marked as “non-novel variants”. Functional effects of nsSNPs were predicted using Polyphen-2 (http://genetics.bwh.harvard.edu/pp2). We downloaded the mutation data for the 15 target genes in 218 TCGA colon/rectal adenocarcinomas (COAD) using the R package “cgdsr” package (CBioPortal, MSKCC) and calculated the frequency of each mutation in the TCGA samples.

### Microsatellite instability (MSI)

The MSI status of the samples was determined as described in our previous studies [28, 60, 61]. Briefly, the extracted tumor and normal matched DNA were used as template in PCR reactions where five microsatellite markers ([62]; *BAT25, BAT26, D17S250, D5S346* and *D2S123*) were used to evaluate the MSI status. PCR products were analyzed in a 3130 ABI GeneScan. Those displaying DNA instability at only one of the markers (including the dinucleotides) were labeled MSI-L, those displaying instability with two or more markers were labeled MSI-H, and those displaying no instability with any of the five markers tested were labeled MSS.

## SUPPLEMENTARY MATERIALS TABLES




