# Supplementary material for: Targeted exome sequencing reveals distinct pathogenic variants in Iranians with colorectal cancer
Source: Oncotarget. 2016 Dec 16;8(5):7852–66. doi: 10.18632/oncotarget.13977 (PMC5341754; doi:10.18632/oncotarget.13977)
Supplement: Supplementary file 1 [file oncotarget-08-7852-s001.pdf]

Targeted exome sequencing reveals distinct pathogenic variants in Iranians with colorectal cancer

Supplementary Materials

Supplementary Tables S1–S12: Summary of validated variants in CRC-associated genes. See Supplementary_Tables_S1–S12
